# Neuromuscular Fatigue Does Not Impair the Rate of Force Development in Ballistic Contractions of Submaximal Amplitudes

**DOI:** 10.3389/fphys.2018.01503

**Published:** 2018-10-24

**Authors:** Gennaro Boccia, Davide Dardanello, Paolo Riccardo Brustio, Cantor Tarperi, Luca Festa, Chiara Zoppirolli, Barbara Pellegrini, Federico Schena, Alberto Rainoldi

**Affiliations:** ^1^NeuroMuscularFunction Research Group, School of Exercise & Sport Sciences, Department of Medical Sciences, University of Turin, Turin, Italy; ^2^CeRiSM Research Center for Sport, Mountain, and Health, University of Verona, Rovereto, Italy; ^3^School of Sport and Exercise Sciences, Department of Neurosciences, Biomedicine and Movement Sciences, University of Verona, Verona, Italy

**Keywords:** central fatigue, peripheral fatigue, explosive strength, rate of force development scaling factor, endurance running

## Abstract

The effect of muscle fatigue on rate of force development (RFD) is usually assessed during tasks that require participants to reach as quickly as possible maximal or near-maximal force. However, endurance sports require athletes to quickly produce force of submaximal, rather than maximal, amplitudes. Thus, this study investigated the effect of muscle fatigue induced by long-distance running on the capacity to quickly produce submaximal levels of force. Twenty-one male amateur runners were evaluated before and shortly after a half-marathon race. Knee extensors force was recorded under maximal voluntary and electrically evoked contractions. Moreover, a series of ballistic contractions at different submaximal amplitudes (from 20 to 100% of maximal voluntary force) was obtained, by asking the participants to reach submaximal forces as fast as possible. The RFD was calculated for each contraction. After the race, maximal voluntary activation, resting doublet twitch, maximal force, and RFD during maximal contraction decreased (-12, -12, -21, and -19%, respectively, all *P*-values < 0.0001). Nevertheless, the RFD values measured during ballistic contractions up to 60% of maximal force were unaffected (all *P*-values > 0.4). Long-distance running impaired the capacity to quickly produce force in ballistic contractions of maximal, but not of submaximal, amplitudes. Overall, these findings suggest that central and peripheral fatigue do not affect the quickness to which muscle contracts across a wide range of submaximal forces. This is a relevant finding for running and other daily life activities that rely on the production of rapid submaximal contractions rather than maximal force levels.

## Introduction

Muscle fatigue can be defined as an exercise-induced decreased capacity to generate maximal force (Gandevia, 2001). Thus, measuring reductions in maximal voluntary contraction force (MVCF) is considered as the most valid and widespread approach to measure muscle fatigue (Millet and Lepers, 2004; Place et al., 2010). It is a sensitive and reliable procedure, which is easy to administer in a variety of research settings (Wilson and Murphy, 1996). Nevertheless, its functional value has been recently questioned (Maffiuletti et al., 2016; Rodriguez-Rosell et al., 2017). Indeed, in many daily and sport activities, the time required to develop maximal force (300 ms or more) is longer than the time available to develop force (Maffiuletti et al., 2016). Therefore, under the time-restricted conditions of short muscle actions, the ability to rapidly produce force is considered functionally more important than maximal force (Maffiuletti et al., 2016; Rodriguez-Rosell et al., 2017). Moreover, it was shown that the capacity to maintain a rapid force production is important in fatigued states, even though this aspect has received little attention in the literature so far (Girard et al., 2015; Morel et al., 2015).

The rate of force development (RFD) reflects the ability to rapidly increase muscle force after the onset of an explosive voluntary contraction (Maffiuletti et al., 2016; Rodriguez-Rosell et al., 2017). RFD has been usually assessed in contractions that require participants to reach as quickly as possible maximal or near-maximal force (usually higher than 80% of maximal force; Folland et al., 2014). Throughout the manuscript we will consider the RFD measured following the above-mentioned procedure as *maximal RFD*. Maximal RFD is likely to be an important determinant of performance in explosive tasks like the shot put (Zaras et al., 2016) or the vertical jump (McLellan et al., 2011). However, it may not reflect the demands of those sports and daily life activities that rely on submaximal muscle contractions, such as endurance running. Indeed, Kulmala et al. (2016) showed that when running at 4.1 m⋅s^-1^, the peak force exerted by knee extensors reached the 63 ± 17% of their maximal force (calculated during hopping task). Thus, in endurance running knee extensors do not exert the maximal force they are capable of, rather they quickly produce a certain amount of force that is needed at each step for the propulsion of the body at a given speed (Kyrolainen et al., 2005; Kluitenberg et al., 2012; Bigouette et al., 2016). For this reason, we suggest that the RFD measured during ballistic contraction of submaximal amplitude could have higher ecological validity than maximal RFD in endurance activities, such as running. Consequently, a distinction should be drawn between ballistic contraction of maximal amplitudes, usually adopted in the literature to calculate the maximal RFD, and ballistic contraction of submaximal amplitudes, which are possibly more relevant for endurance running. While it has been demonstrated that running-based fatiguing protocols impair the maximal RFD (Oliveira et al., 2013; Boccia et al., 2017a, 2018), it is unknown to what extent muscle fatigue may impair the ability to quickly produce forces of submaximal amplitudes.

To this aim, a protocol usually adopted to calculate the so-called RFD scaling factor (RFD-SF) provides an appealing approach (Bellumori et al., 2011, 2013; Casartelli et al., 2014; Djordjevic and Uygur, 2017). The protocol consists in a series of quick and fast (i.e., ballistic) contractions performed with different submaximal amplitudes (namely from 20 to 100% of MVCF) (Freund and Budingen, 1978; Ghez and Vicario, 1978; Wierzbicka et al., 1991; Klass et al., 2008). In each ballistic contraction the individuals are asked to roughly reach a given submaximal force as fast as possible (the emphasis is on the quickness of the contraction rather than on the accurateness). The RFD measured in each contraction thus quantify the capacity to quickly produce submaximal force (Bellumori et al., 2011, 2013; Casartelli et al., 2014; Djordjevic and Uygur, 2017).

In the aforementioned protocol, the RFD-SF consists in the slope of the linear relationship between the peak force and the peak RFD obtained in each ballistic contraction (Bellumori et al., 2011, 2013; Casartelli et al., 2014; Djordjevic and Uygur, 2017). This relationship has been investigated since the late 1970s (Freund and Budingen, 1978; Ghez and Vicario, 1978). The consistent finding since then was a linear increase in RFD at rising force amplitudes: i.e., the higher the force required, the quicker the contraction (Freund and Budingen, 1978; Ghez and Vicario, 1978; Wierzbicka et al., 1991; Van Cutsem et al., 1998). While the physiological mechanisms underpinning the RFD-SF are far from being elucidated, the available studies suggest that RFD-SF measurement may inform about the important features of movement initiation and quickness of force production (Wierzbicka et al., 1991; Bellumori et al., 2011). Indeed, the RFD-SF is known to be lower in older adults compared to young adults (Klass et al., 2008; Bellumori et al., 2013) and also in people with Parkinson’s disease compared to healthy people (Wierzbicka et al., 1991). Furthermore, RFD-SF increases after a period of power training both in young and elderly people (Van Cutsem et al., 1998; Bellumori et al., 2017), its improvement being related to an increase in motor units discharge rate (Van Cutsem et al., 1998; Klass et al., 2008). The fact that RFD-SF can reflect neural factors was furthermore substantiated by the study of Van Cutsem and Duchateau (2005). They showed that performing ballistic contractions from a sustained contraction, compared to a resting state, decreased the instantaneous motor unit discharge rate and the RFD, thereby reducing the RFD-SF (Van Cutsem and Duchateau, 2005). Beyond neural factors, biochemical and mechanical muscle properties such as muscle fiber type, fiber shortening velocity, and elastic properties of the muscle-tendon unit (Maffiuletti et al., 2016) are known to influence RFD, even though their effect on RFD-SF has not been investigated so far. However, despite the neuromuscular correlates are not clear, RFD-SF has been considered to be an appealing measure to compare individuals and populations independently to their strength (Bellumori et al., 2011), since it is not affected by maximal force capacity. It is well known that both central and peripheral mechanisms contribute to impair maximal RFD after fatiguing exercise (Buckthorpe et al., 2014; Boccia et al., 2016, 2018; Maffiuletti et al., 2016), but the impact of fatigue on RFD-SF has not been yet investigated.

Therefore, the aims of the study were to assess if muscle fatigue induced by long-distance running would impair (1) the RFD exerted in ballistic contractions of submaximal amplitudes; and/or (2) the RFD-SF of knee extensor muscles. We hypothesized that a half-marathon race decreases the RFD in ballistic contractions of submaximal amplitudes and the RFD-SF.

## Materials and Methods

### General Overview

**FIGURE 1:**
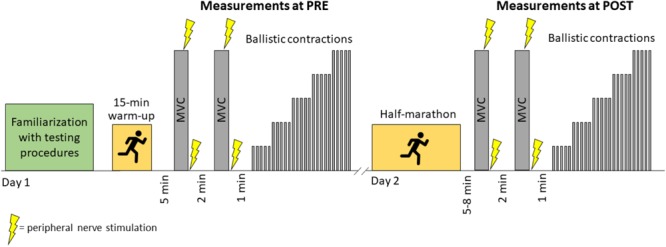
Schematic representation of experimental procedures. MVC, maximal voluntary contraction.

The study was performed during a scientific event called *Run For Science*, hosted by the University of Verona (Italy) in April 2017, for details see (Lippi and Schena, 2017). In this event, participants competed in an official half-marathon race certified by Italian Track and Field Federation. The day of the race the weather was sunny, with no wind, the air temperature was 18° and humidity 70% and these conditions were quite stable along the duration of the event. The start waves were assigned to participants based on the individual estimated race time, to avoid many participants reaching simultaneously the testing station. The assessments consisted in a series of voluntary and electrically evoked contractions of the knee extensors. Participants were involved in two measurement sessions: the first was performed the day before the race (PRE), and the second shortly after the race (POST). A schematic representation of the experimental procedures is reported in the Figure 1. Participants were instructed to refrain from performing strenuous physical activity in the 24 h before the first experimental session. They were also asked to refrain from consuming caffeine in the 24 h before the first experimental session and before the race. During this first session, participants were familiarized with the maximal voluntary contractions, peripheral nerve electrical stimulation, and RFD-SF protocol. For that purpose, participants repeated two or three trials of the test procedures until they were able to produce consistent results. Before the neuromuscular testing at PRE, participants performed a warm-up consisting of 15 min of outdoor running at an incremental intensity from 75 to 90% of predicted maximal heart rate (Bishop, 2003). In the POST session the neuromuscular assessment started within 5 – 8 min after the race. A researcher was positioned at the finishing line to conduct the runners to the testing site, an indoor room located about 50 m from the finishing line. The testing session at POST lasted about 6 min.

### Participants

Twenty-three amateur male runners (age 45 ± 9 years, body mass 73 ± 11 kg, height 175 ± 8 cm) participated in this study. They were recruited through printed and electronic media, advertising the possibility to be a subject for scientific study within the event *Run for Science* (Lippi and Schena, 2017). Inclusion criteria were to be regularly engaged in recreational running (mean training regimen of 220 min/week), to have finished a half-marathon in the previous 2 years, and to be free from clinical evidences of cardiovascular, neuromuscular, or joint diseases. All participants provided their written informed consent before participation in the experiments. The study was approved by the local Ethical Committee (Department of Neurological and Movement Sciences, University of Verona) and performed in accordance with the Helsinki Declaration.

### Data Acquisition

For the assessment of knee extensor muscles, participants were seated on a custom-made chair with straps fastened across their chest and hips to avoid undesired lateral and frontal trunk displacements. During the testing, participants’ knee and hip were flexed at 90° from full extension and they were instructed to maintain the arms crossed on the chest. The knee extensors mechanical response was recorded with a strain gauge load cell (546QD- 220 kg; DSEurope, Milan, Italy), fixed with non-compliant straps to the leg at the level of the external malleolus. All measurements were taken from the participants’ right limb (which was the dominant limb for 21 out of 23 participants). Throughout the measurement session, visual feedback of the force output was provided as a real time signal displayed on a computer screen.

Electromyographic (EMG) signals were recorded from *vastus medialis* and *vastus lateralis* muscles with pairs of silver chloride (Ag/AgCl) circular (recording diameter of 10 mm) surface electrodes (Kendall Meditrace 100) aligned to the muscle fibers according to guidelines (Rainoldi et al., 2004), with an interelectrode (center-to-center) distance of 20 mm. The force and EMG signals were sampled at 2048 Hz and converted to digital data with a 12-bit A/D converter (EMG-USB, OT Bioelettronica, Turin, Italy). Force signal was conditioned with a moving averaging window of 0.01 s to remove noise. EMG signals were amplified with a gain of 500, band-pass filtered with a bandwidth frequency between 20 and 450 Hz (4th-order, zero lag Butterworth).

### Electrical Stimulation

A constant current stimulator (Digitimer DS7A, Hertfordshire, United Kingdom) was used to deliver a square-wave stimulus of 1 ms duration with maximal voltage of 400 V. The cathode (2-cm diameter, Meditrace 100 Kendall; Tyco, Markham, ON, Canada) and the anode (5 × 10 cm; Compex, Ecublens, Switzerland) were placed over the femoral nerve at the femoral triangle level beneath the inguinal ligament and on the lower part of the gluteal fold opposite to the cathode, respectively. Supra-maximal stimulation intensity level was determined by increasing the applied current until a plateau in maximal twitch was obtained. The stimulation intensity (range: 120 – 320 mA) was then increased by 20%, to ensure supra-maximal stimulus (120% of optimal intensity) and kept constant throughout the experiment. The optimal placement for stimulation electrodes were marked on the skin during PRE to ensure the same position during POST.

### Procedure

The neuromuscular test comprised two sets of the following: one maximal voluntary contraction (duration of 4 s) of the knee extensors with super-imposed supra-maximal paired stimuli (doublet) at 100 Hz and followed (2 s intervals) by paired stimuli at 100 Hz (generating a *high frequency* doublet twitch, Db100), delivered in resting state. Then a paired stimulation at 10 Hz (generating a *low frequency* doublet twitch, Db10) and a single stimulation were delivered, interspaced by 5 s, to evoke M-wave and a resting twitch (Tw). This was followed by 2 min of rest. If the difference between the two MVCF was higher than 5%, a third set of this procedure was performed. Standardized verbal encouragements were provided to the participants during the execution of maximal voluntary contractions.

**FIGURE 2:**
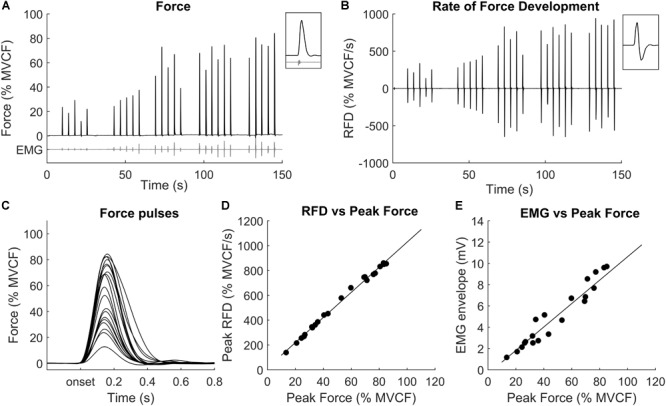
Representative example of a set of ballistic contractions performed across a range of submaximal amplitudes. **(A)** Force signals recorded during 5 or 6 ballistic contraction for each force level; **(B)** RFD signals (first derivative of force); **(C)** superimposed force signals of each ballistic contraction; **(D)** each point represents the peak RFD (*y* value) and the peak force (*x* value) achieved in each contraction; **(E)** each point represents the electromyographic (EMG) envelope (*y* value) and the peak force (*x* value) achieved in each contraction.

The RFD-SF protocol started 1 min after the last maximal voluntary contraction. Similar to the methods used by others (Freund and Budingen, 1978; Klass et al., 2008) the RFD-SF relationship was computed from sets of several fast and quick isometric contractions (ballistic contraction) performed across a full range of amplitudes (Figures 2A–C). Participants were instructed to produce isometric ballistic contractions in order to achieve peak force as quickly as possible and then relax instantly (Figure 2C). Participants performed five consecutive contractions at five different intensities presented in an ascending order (20, 40, 60, 80, and 100% MVCF). The timing of contractions was cued by digital stimuli 4 s apart. Participants were explicitly instructed not to target the force levels requested because targeting slows the rate of force production (Gordon and Ghez, 1987). Instead, they were asked to produce fast contractions with peak forces reaching approximately the area around the target line. As previously suggested (Bellumori et al., 2011, 2013; Casartelli et al., 2014), in the familiarization session participants practiced until they felt comfortable with the task and could perform discrete ballistic contractions as instructed.

### Data Analysis

#### Mechanical Signals

All data were analyzed by custom-written software in MATLAB R2015a (Mathworks, Natick, MA, United States). The amplitude of the resting doublet and single twitches (Db100, Db10, and Tw) were analyzed and the average value computed from the two sets was considered. The level of voluntary activation (VA) during each maximal voluntary contraction was calculated as VA(%) = 100(1 - interpolated doublet/Db100) × 100 (Merton, 1954). A correction was consistently applied to this equation when the superimposed doublet was elicited slightly before or after the actual peak force during a maximal voluntary contraction (Strojnik and Komi, 1998). The Db10:Db100 ratio was used as a surrogate measure of low-frequency fatigue, which is usually associated with a failure in the excitation-contraction coupling (Verges et al., 2009; Millet et al., 2011).

To calculate the RFD-SF, the force signal was firstly pre-processed using an overlapping moving window of 0.1 s (Bellumori et al., 2011). If a countermovement (i.e., a visible drop in force) was performed before the force onset, the contraction was discarded from the analysis. Then, the first derivative of the force signal was computed to obtain the RFD (N/s, see Figure 2B). For each subject and all contractions, peak force and peak RFD (which is local maximum of the RFD signal) were computed and plotted to obtain a linear regression (Figure 2D). Outliers were removed using the Cook distance methodology (Cook, 2000). The linear regression parameters (slope, y-intercept, *R*^2^) were then calculated and considered as outcomes. In 14 occasions out of 46 (23 participants for two sessions, i.e., PRE and POST), the relationship between peak forces and peak RFD was not linear for the whole range of submaximal contractions. More precisely, it was linear from 0 to about 80–90% of the maximal force and then showed a logarithmic behavior from about 80–90% to the maximum. In these occasions, biphasic regression explained more variation than a linear regression between force and RFD (Linden, 2015). The breakpoint for this interrupted regression was calculated (Linden, 2015) and the coefficients for the first part of linear regression, i.e., up to the breakpoint, was reported. To compare the absolute values of RFD between subjects, the peak force and the peak RFD were then normalized to the MVCF (Richartz et al., 2010) obtained at PRE. Notably, the RFD data at POST were normalized with respect to the MVCF at PRE as well. This choice is important because we wanted to compare the RFD capacity between PRE vs. POST when targeting the same absolute torque, which was the first experimental question of this study. Normalizing the RFD for the MVCF at POST would have prevented to answer this question.

To assess if RFD exerted in rapid contractions of submaximal amplitude changed after the race, we compared the RFD produced at the same absolute force level between PRE and POST. Since it is virtually impossible to have two ballistic contractions with the same amplitude in PRE and POST, we decided to evaluate the linear regression between RFD and force at predetermined force intervals across the whole available range of forces. To do this, the regression line of the RFD-SF was evaluated from 10% of the maximal force at PRE, to the highest available peak with 10% intervals (i.e., 10%, 20%, … 100% of the MVCF at PRE).

Moreover, the *maximal RFD* was considered as the RFD recorded during the contraction that presented the highest RFD.

#### EMG Signals

M-wave properties, that is M-wave area (M_AREA_), peak-to-peak amplitude (M_AMPLITUDE_) and duration (M_DURATION_) were measured from the EMG response obtained by single stimulations at rest. The root mean square (RMS) of EMG signals was calculated over a 500 ms epoch centered at the peak force of the maximal voluntary contraction with the greatest force (RMS_MV C_). The RMS_MV C_/M_AMPLITUDE_ ratio was calculated as an index of muscle activation independently by changes in muscle excitability (Millet and Lepers, 2004). As recently suggested (Balshaw et al., 2017), the values coming from *vastus medialis* and *vastus lateralis* were averaged to increase within-participant reliability.

The EMG amplitude during RFD protocol was calculated as the envelope of the rectified EMG signal calculated in the 200-ms epoch before the RFD peak of each contraction (Figure 2E). The EMG value calculated as such may be considered as the overall amount of EMG activity that preceded the RFD peak. Since in this study the RFD peaks occurred at about 60–90 ms after the force onset, the length of the epoch ensured that the onset of EMG activity (which was not calculated) was included even when considering an electromechanical delay of 20 – 60 ms. The EMG amplitude calculated as such was normalized to the M_AMPLITUDE_ to provide an index of muscle activation, independently by changes in muscle excitability.

### Statistical Analysis

Statistical analysis was performed with MATLAB R2015a. Data are presented as mean ± standard deviation (SD). Kolmogorov–Smirnov normality test was used to assess distributions normality. If the data were not normally distributed, they were log-transformed before statistical analysis and back-transformed to obtain descriptive statistics. Paired, two-tailed Student’ *t*-tests were used to compare the RFD-SF linear regression parameters, mechanical, and EMG variables between PRE vs. POST. The level for statistical significance was set to *P* < 0.05. Differences between PRE vs. POST were reported in absolute and percent values, the precision of estimates for absolute values was indicated with 90% confidence interval (CI). The magnitude of the difference was calculated as Cohen’s *d* effect size. Threshold values for effect size statistics were: <0.2, trivial; >0.2, small; >0.6, moderate; >1.2 large; >2.0, very large (Batterham and Hopkins, 2006).

## Results

Out of the 23 initially recruited participants, two individuals did not tolerate the peripheral nerve stimulation, thus data are reported for 21 participants. Their mean race time was 112 ± 11 min.

The results of the voluntary and evoked contractions are reported in Table 1. Briefly, MVCF and muscle activation during the maximal voluntary contraction (RMS_MV C_) decreased in POST with respect to PRE (-21 ± 10% and -22 ± 33%, respectively). Furthermore, decreases in voluntary activation (VA: -12 ± 8%), muscle contractile properties (Db100: -12 ± 8%; Db10:Db100: -9 ± 10%), and muscle excitability (M_AMPLITUDE_: -4 ± 15%; M_AREA_: -7 ± 13%) were found. Maximal RFD and the associated EMG amplitude (RMS_RFD_) decreased after the race (-19 ± 18% and -14 ± 12%, respectively).

**Table 1:** Voluntary and electrically evoked responses before and after the race.

	PRE		POST		Difference	90% CI		Percent difference		Effect size (Cohen’s *d*)	*P*-value
	Mean	*SD*	Mean	*SD*		Lower bound	Upper bound	Mean	*SD*	
MVCF (N)	524.8	144.7	426.3	159.3	–98.5	–110.6	–86.4	–20.6	9.6	0.64	<0.0001
Maximal RFD (N/s)	3747.7	801.2	3160.9	852.5	–586.8	–735.5	–438.1	–21.5	18.1	0.71	<0.0001
VA (%)	91.1	7.2	83.3	10.2	–7.7	–9.6	–5.8	–8.7	6.5	0.81	<0.0001
RMS_MV C_/M_AMPLITUDE_	5.5	1.9	4.0	1.4	–1.5	–1.9	–1.1	–24.8	16.9	0.90	<0.0001
RMS_RFD_ (mV)	1.82	0.45	1.48	0.41	–0.29	–0.46	–0.12	–27.2	45.8	0.79	0.0188
Db100 (N)	250.5	43.7	226.4	47.8	–24.2	–31.6	–16.8	–9.8	8.8	0.53	<0.0001
RFD_Db100_ (N/s)	9244.4	2637.7	7580.8	2312.4	–1663.5	–2181.0	–1146.1	–16.8	16.4	0.67	<0.0001
Db10 (N)	224.9	43.6	186.0	51.1	–38.8	–48.8	–28.9	–17.7	13.0	0.81	<0.0001
Db10:Db100 (%)	89.8	9.2	81.8	12.8	–7.9	–10.8	–5.1	–9.0	9.2	0.63	0.0004
Single twitch (N)	157.1	34.1	133.5	36.4	–23.7	–31.0	–16.4	–15.1	13.5	0.69	<0.0001
M_DURATION_ (ms)	20.9	1.1	20.9	1.2	0.0	–0.3	0.3	0.2	4.5	0.00	0.8501
M_AMPLITUDE_ (mV)	2.70	0.63	2.52	0.54	–0.17	–0.29	–0.06	–4.7	14.6	0.31	0.0266
M_AREA_ (mV⋅ms)	0.374	0.091	0.346	0.075	–0.028	–0.043	–0.012	–5.9	12.6	0.33	0.0112

*MVCF, maximal voluntary contraction force; Maximal RFD, rate of force development recorded during the contraction that presented the highest RFD; Maximal RFD_*normalized*_, Maximal RFD normalized to MVCF; VA, voluntary activation; M_*AMPLITUDE*_, peak-to-peak amplitude of the M-wave; RMS_*MVC*_/M_*AMPLITUDE*_, root mean square of electromyographic signals recorded during the MVC normalized to M_*AMPLITUDE*_; RMS_*RFD*_, root mean square of electromyographic signals recorded during the maximal RFD normalized to M_*AMPLITUDE*_; Db100, twitch evoked by the doublet at 100 Hz; Db10:Db100, ratio between the twitches evoked with doublets at 10 to 100 Hz; M_*DURATION*_, duration of the M-wave; M_*AREA*_, total area of the M-wave.*

**FIGURE 3:**
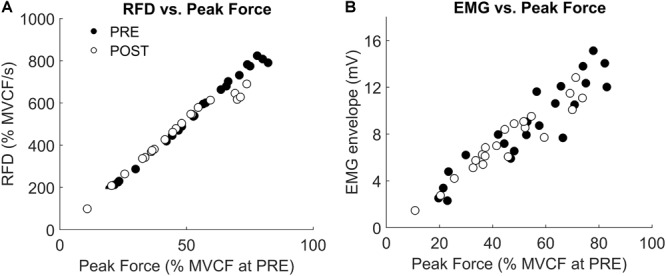
Representative example of **(A)** rate of force development (RFD) and **(B)** electromyographic (EMG) envelope recorded before (PRE) and after (POST) half marathon in a set of ballistic contraction performed across a range of submaximal amplitudes. Force data are normalized with respect to the maximal voluntary contraction force (MVCF) recorded at PRE.

The results of the RFD-SF are reported in Table 2. Briefly, all indices of the RFD-SF were unaffected by the race. Representative example of the relationship between RFD and force for PRE and POST is reported in Figure 3A. It can be seen that, in this specific participant, the RFD values at POST are substantially unchanged with respect to PRE up to the 60% of MVCF. This is confirmed by the evaluation of regression lines reported in the following paragraph.

**Table 2:** Parameters of the linear regression between force and rate of force development achieved during the ballistic contractions before and after the race.

	PRE		POST		Difference	90% CI		Percent difference		Effect size (Cohen’s *d*)	*P*-value
	Mean	*SD*	Mean	*SD*		Lower bound	Upper bound	Mean	*SD*		
Slope (RFD-SF)	7.67	1.88	7.74	2.22	0.07	–0.55	0.43	0.9	1.5	0.03	0.8257
y intercept	396	402	368	389	–28	–67	112	–7.1	6.5	0.07	0.6522
*R*^2^	0.97	0.02	0.96	0.03	–0.01	–0.01	0.02	–1.0	2.0	0.39	0.1165

*PRE, before the race; POST, after the race; RFD-SF, rate of force development scaling factor. See the Section “Materials and Method” for description of the regression line.*

**FIGURE 4:**
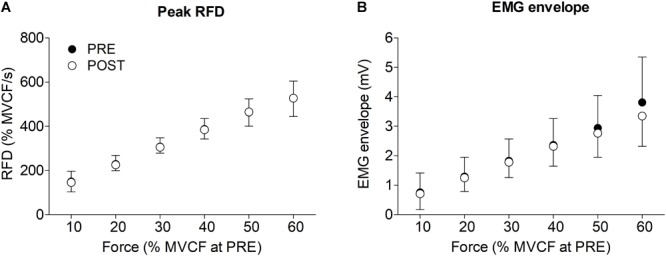
Mean and standard deviation of **(A)** rate of force development (RFD) and **(B)** electromyographic (EMG) envelope before (PRE) and after (POST) half marathon. The values are reported from 10 to 60% of maximal voluntary contraction force (MVCF) recorded before the race (see methods).

The regression line was evaluated at PRE and POST from 10 to 70% of the MVCF at PRE. This was done because the decline in MVCF at POST made the force higher than 70% of MVCF unattainable for all participants. However, not every participant reached at POST the 70% of MVCF obtained at PRE. Thus, for 60 and 70% of MVCF the regression line was evaluated for only 12 and 6 participants, respectively. The RFD calculated across the available range (10–70% of MVCF at PRE) did not change between PRE and POST (all *P*-values > 0.4, Figure 4A). The EMG amplitude calculated in the same range did not change as well (see Figure 3B for a representative example and Figure 4B for group level results).

## Discussion

This study aimed to assess the effect of muscle fatigue induced by a half-marathon on the capacity to quickly contract leg muscles across a wide range of force levels production. The linear relationship existing between RFD and peak force, named RFD scaling factor, did not change as a consequence of fatigue. While the RFD exerted during short and fast contraction of maximal amplitudes decreased, the RFD exerted in short and fast contractions of submaximal amplitude was unaffected by fatigue. Evaluating the effects of fatigue on quick contractions of submaximal amplitudes has been mostly overlooked in the literature and it constitutes the innovation of this study. We advocate that this is relevant for endurance running and for many other sports and daily life activities which rely on quick contractions of submaximal amplitudes.

### Central and Peripheral Fatigue

Shortly after the half-marathon, the maximal force decreased by ≈21%, confirming that the prolonged run induced moderate muscle fatigue in knee extensors. This is in line with our previous studies where we also found a moderate decrease (ranged 11–24%) in maximal force after half-marathons performed in similar experimental conditions (Boccia et al., 2017a,b, 2018). The strength loss is a common finding after endurance running in ecological race conditions: it increases non-linearly with the duration of exercises and it may vary between 8 and 41% (Millet and Lepers, 2004; Place et al., 2010; Giandolini et al., 2016).

The decrease in maximal force was associated with a decrease of similar extent in EMG amplitude normalized to M-wave amplitude (RMS_MV C_/M_AMPLITUDE_ ≈22%). While EMG amplitude *per se* cannot be used to measure the neural drive to the muscles (Martinez-Valdes et al., 2018), a decrement in the EMG amplitude normalized to the M-wave amplitude can be reasonably attributed to central mechanisms (Millet and Lepers, 2004). Furthermore, participants underwent a moderate decrease of voluntary activation (≈12%). Together these findings indicated a reduced capacity to voluntarily activate the knee extensors in maximal contraction and reflect the presence of central fatigue. Central fatigue constitutes an important limitation to performance and is the major cause of the maximal force decrease induced by a prolonged whole body exercise such as running (Saldanha et al., 2008). Central fatigue could reflect the existence of a suboptimal neural drive output from the motor cortex (Taylor et al., 2006). It may nevertheless be located at spinal level (McNeil et al., 2011) and mediated by group III–IV muscle afferents (Sidhu et al., 2017). The amount of central fatigue depends on many exercise parameters, such as intensity, duration, and contraction modality, among others (Taylor et al., 2016). While a detailed discussion of the mechanisms regulating the amount of central fatigue is beyond the scope of this paper, it is evident in the literature that central fatigue is overall greater for extreme-duration running (Millet and Lepers, 2004; Martin et al., 2010; Temesi et al., 2014) rather than for shorter, more intense bouts like half-marathons (Ross et al., 2010; Boccia et al., 2018).

In our investigation, there was also an evidence of peripheral fatigue, as indicated by a small decrease in the doublet amplitude of about 12%. This result is in line with previous studies regarding a race of similar duration (Place et al., 2004; Boccia et al., 2018). The reduction in electrically evoked force can be attributed both to a decrease in sarcolemma membrane excitability (Rodriguez-Falces and Place, 2018), as evidenced by small decrease of M wave amplitude (≈4–7%), and to the impairment of the excitation-contraction coupling mechanism, as evidenced by the moderate decrement of Db10:Db100 ratio (≈9%) (Place et al., 2010). In particular, the latter represents the disproportionate loss of force at low compared with high frequencies of electrical stimulation: it is termed low-frequency fatigue and it is most likely due to an impairment in calcium release or reuptake from the sarcoplasmic reticulum (Millet et al., 2011; Janecki et al., 2016).

For sure, the delay (5 – 8 min) between the end of the race and the beginning of the testing allowed a partial recovery in both central (Gruet et al., 2014; Mira et al., 2017) and peripheral fatigue (Froyd et al., 2013). Indeed, it has been shown that substantial central and peripheral recovery occurs in the first 5 min after task completion (Carroll et al., 2017). Thus, during final part of the race, that is when fatigue possibly influenced mainly the running performance, the central and peripheral fatigue were likely greater than the ones recorded in the measurements. Therefore, the impairment that we found in maximal force and RFD (discussed below) were likely underestimated as well. However, since the measurements of central and peripheral fatigue were concomitant (within few seconds) to the maximal force and RFD assessment, the magnitude of fatigue reported here is the level of fatigue that influenced the maximal force and RFD measurements.

### RFD in Contractions of Maximal and Submaximal Amplitude

The maximal RFD moderately decreased after the race (≈19%). This is in line with some previous studies investigating prolonged running (Kelly et al., 2011; Oliveira et al., 2013; Boccia et al., 2017a, 2018). The muscle activation (EMG amplitude normalized to M-wave amplitude) measured during the execution of maximal RFD moderately decreased (≈14%), suggesting that the impairment in RFD can be attributed, at least in part, to a suboptimal neural drive to the muscles (Maffiuletti et al., 2016). However, the observed peripheral fatigue may have influenced the maximal RFD as well (Maffiuletti et al., 2016).

The most interesting result of this study was that despite the presence of significant amount of central and peripheral fatigue, the capacity to quickly produce ballistic contraction of submaximal amplitude remained unaffected. Indeed, the RFD recorded during submaximal tasks, was maintained after the half-marathon. Because of the drop in maximal force, after the race the participants were not able to reach force levels higher than 70% of the maximal force produced before the race. However, up to that level, the RFD values recorded in fatigued state were similar to those recorded in fresh state. Interestingly, a previous study (Kulmala et al., 2016) found that the peak force exerted by the knee extensors when running at 4.1 m⋅s^-1^ reached the ≈63% of maximal force, which is below the limit where the RFD was unaltered by fatigue in the present study. Together, these findings can explain why a recent meta-analysis showed that muscle fatigue did not significantly change the ground reaction force active peak and loading rate in running (Zadpoor and Nikooyan, 2012). To understand if this non-different RFD was associated to changes in muscle activation, the EMG amplitude, normalized to the M-wave amplitude, was calculated from the beginning of the contraction to the peak RFD of each ballistic contraction. An increase in EMG amplitude would mean that the central nervous system should increase muscles activation to produce the same RFD, thus suggesting a decrease in neuromuscular efficiency. However, the EMG amplitude did not change after the race (Figures 3B, 4B) suggesting that to produce RFD in contractions of submaximal amplitudes the central nervous system does not have to increase the neural drive to the muscles. Therefore, the RFD in fatigued state was produced as effectively as in fresh state for a wide range of submaximal contractions. Runners are accustomed to repeatedly cope with the transient of the vertical ground reaction force within the first 100–150 ms of stance (Lieberman et al., 2010). Thus, this finding may be attributed to the specific population of habitual runners recruited in this study. Further studies are needed to understand if different population and/or fatiguing exercises would give different results.

The fact that only the maximal RFD was affected by fatigue poses interesting questions about the effects of fatigue on muscle performance. As the RFD recorded during maximal contractions is higher than the RFD calculated during submaximal contractions it is possible to hypothesize that fatigue affected the RFD only when the increase of force was at its highest possible rate. When performing maximal explosive contractions high-threshold motor units are recruited since the beginning of the contraction, at very low force level (Heckman and Enoka, 2012). However, this is not true when the contraction is slower and the transient of force is less steep (Heckman and Enoka, 2012). Together, this finding may allow to speculate that central fatigue results in inability to activate high-threshold motor units, while preserving the capacity to activate low threshold motor units, thus maintaining the capacity to produce less steep RFD. The same can be said for peripheral fatigue: while the lowered contractile properties may reduce the capacity to produce maximal RFD (Jones, 2010), the residual contractile properties seemed to be enough to produce less steep RFD. While we still agree on the goodness of measuring the maximal RFD as an index of muscle fatigue, the decrease of maximal RFD unlikely has a direct effect on mechanics of long duration exercises. In an attempt to measure neuromuscular adjustments that mechanistically modulate performance (Enoka and Duchateau, 2016), we propose that the measurement of RFD on quick contractions of submaximal amplitude may be more relevant than maximal RFD in endurance context.

### RFD-SF

Since RFD-SF was generally associated with features of muscle contraction quickness, we hypothesized that this parameter should be altered by fatigue, but this was not the case (Figure 3A and Table 2). As it can be seen from the data of a representative participant reported in Figure 3A, the linear regressions before and after the running were closely similar up the 60–70% of maximal force recorded before the race. Fatigue lowered the maximal available force, consequently limiting the extent to which the regression extends rightward but did not change the slope of the regression. This result, obtained with an original experimental approach, confirmed that the RFD-SF is independent from muscle strength (Bellumori et al., 2011; Casartelli et al., 2014; Djordjevic and Uygur, 2017). Furthermore, it demonstrated for the first time that RFD-SF is independent from the fatigue induced by a prolonged running race. Even though maximal muscle strength decreased by 21% after the race, the RFD-SF remained unaltered. Furthermore, this result helps to shed light on the neuromuscular correlates of RFD-SF, by excluding moderate muscle fatigue from the potential determinants. Since we observed a moderate decrease in voluntary activation and muscle contractile properties, this result suggests that a moderate impairment in both central and peripheral neuromuscular properties did not affect the RFD-SF. However, future studies should investigate if different exercise modality and/or more severe muscle fatigue may affect the RFD-SF.

While the slope of the regression between force and RFD is usually adopted as the main parameter, the intercept of this regression may also be of interest. Indeed, fatigue could potentially shift downward the regression, without modifying its slope. Contrary to the expectation, the intercept of the linear regression was unaffected by fatigue as well (Table 2). Together, these results suggest that (at least across submaximal forces) the relationship between force and RFD remained unaltered in fatigued condition. Hence the RFD-SF cannot be used as an index of fatigue on the basis of the herein findings. However, since it can provide a measure of contraction quickness independently from the decrease in maximal force that can be expected in presence of fatigue, potential usefulness of RFD-SF in the context of muscle fatigue is still to be determined.

### Limitations

The results of this study were specific to the type of exercise adopted to induce fatigue, i.e., prolonged running of about 2 h. It is not possible to infer how the RFD and RFD-SF would behave when adopting different fatiguing protocols. Furthermore, the participants were habitual recreational runners, thus our results cannot be generalized to untrained population.

While the EMG activity during the ballistic contractions was unaffected by fatigue, it is reasonable to think that the recording of mechanomyogram would have provided insightful information about the mechanical determinants of this type of contraction. Indeed, mechanomyograms features were reported to be sensitive to fatigue even when the EMG response was unaltered (Perry-Rana et al., 2002) and were useful to identify possible mechanical adjustments occurring across a series of brief isometric contraction from 10 to 100% of maximal force (Beck et al., 2004). For these reasons, future studies are warranted to include mechanomyogram analysis to further elucidate the effect of fatigue on this type of contractions.

Another main limitation was that while we measured the *performance fatigability* (i.e., the mechanical output) of the participants, we did not measure the *perceived fatigability*, see (Enoka and Duchateau, 2016) for review. Indeed, while we demonstrated that to produce ballistic contraction of submaximal amplitudes the RFD and the associated muscle activation were the same after the race, this does not mean that participants perceived the same effort to produce the ballistic contractions. Thus, future studies are warranted to understand if this preservation of RFD in ballistic contractions would be associated to possibly higher perceived effort.

## Conclusion and Perspectives

To summarize, long-distance running of about 2 h affects the capacity to quickly produce force during ballistic contractions of maximal, but not submaximal, amplitudes. The RFD scaling factor was also unaffected after the race. Overall, these findings suggest that central and peripheral fatigue did not affect the quickness of contraction across a wide range of submaximal force levels. This is a relevant finding because running, as many other endurance sport and daily life activities, relies on fast and brief contractions aimed to produce submaximal, rather than maximal, force levels. Future studies should assess if this capacity may be affected by fatiguing task of different intensity, length and contraction modality.

## Author Contributions

GB and DD: conceptualization. GB, DD, PRB, CZ, CT, LF, and BP: methodology. GB, DD, PRB, CZ, CT, LF, BP, FS, and AR: investigation. GB and PRB: formal analysis. GB: writing – original draft. GB, DD, PRB, CZ, CT, LF, BP, FS, and AR: writing – review and editing. FS and AR: supervision.

## Conflict of Interest Statement

The authors declare that the research was conducted in the absence of any commercial or financial relationships that could be construed as a potential conflict of interest.
